# Multi-Branch Attention Learning for Bone Age Assessment with Ambiguous Label

**DOI:** 10.3390/s23104834

**Published:** 2023-05-17

**Authors:** Bishi He, Zhe Xu, Dong Zhou, Yuanjiao Chen

**Affiliations:** School of Automation (School of Artificial Intelligence), Hangzhou Dianzi University, Hangzhou 310018, China

**Keywords:** computer vision, medical image processing, bone age assessment, ambiguous label, image content understanding

## Abstract

Bone age assessment (BAA) is a typical clinical technique for diagnosing endocrine and metabolic diseases in children’s development. Existing deep learning-based automatic BAA models are trained on the Radiological Society of North America dataset (RSNA) from Western populations. However, due to the difference in developmental process and BAA standards between Eastern and Western children, these models cannot be applied to bone age prediction in Eastern populations. To address this issue, this paper collects a bone age dataset based on the East Asian populations for model training. Nevertheless, it is laborious and difficult to obtain enough X-ray images with accurate labels. In this paper, we employ ambiguous labels from radiology reports and transform them into Gaussian distribution labels of different amplitudes. Furthermore, we propose multi-branch attention learning with ambiguous labels network (MAAL-Net). MAAL-Net consists of a hand object location module and an attention part extraction module to discover the informative regions of interest (ROIs) based only on image-level labels. Extensive experiments on both the RSNA dataset and the China Bone Age (CNBA) dataset demonstrate that our method achieves competitive results with the state-of-the-arts, and performs on par with experienced physicians in children’s BAA tasks.

## 1. Introduction

Human growth and development have an objective judgment standard, i.e., bone age, except for physical age. The early diagnosis of growth and development anomalies in teenagers based on bone age can not only estimate growth but also detect potential endocrine diseases, thus allowing intervention measures to be adopted timely [1,2]. Bone age assessment (BAA) is usually implemented by radiologists by examining left-hand X-ray images. Currently, the most common manual methods are Greulich–Pyle (GP) atlas and Tanner Whitehouse 2 (TW2) scoring method. For the GP atlas method [3], doctors determine bone age by searching the standard reference image set for the most similar image to the X-ray image of the patients’ hand. The TW2 method [4] recognizes the specific regions of interest (ROIs) of wrist and finger bones, then scores each ROI according to development degree, and finally combines the scores to calculate bone age. However, both the GP atlas and TW2 scoring methods suffer from some limitations in BAA. First, these two methods have strong subjectivity and are easily interfered by doctors’ subjective judgment. Moreover, they involve high requirements on professional levels of doctors, and bone age can be determined accurately only if provided with long-term training and experience. Lastly, both methods are time- and labor-consuming. Currently, the GP atlas is the preferred method because the TW2 method requires determining the grade of each RoI, which increases the workload of physicians.

Deep learning (DL) has been used with tremendous success in a variety of medical image analysis tasks, such as pulmonary nodule detection [5], chest X-ray diagnosis [6], and intracranial hemorrhage detection [7]. Accordingly, scholars have proposed a series of DL techniques for BAA using the open BAA dataset Radiological Society of North America (RSNA) and achieved excellent performance. DL-based BAA methods can generally be classified into two categories: single-stage and multi-stage structures. The former one pretrains the networks such as VGG [8], ResNet [9], or Inception [10] on ImageNet [11] and then predicts the bone age directory by adding a regression layer at the end of the network. The latter first segment the hand object from original images by employing an ROI-based method with additional processing, then detects and extracts bone part regions with human prior knowledge, and finally predicts bone age through a regression layer. In comparison with the single-stage structure, the multi-stage structure achieves better performance. Although previous related research have confirmed the feasibility of deep learning in BAA with outstanding performance, they mainly train, verify, and test their network on RSNA or private dataset. None of these studies have evaluated the accuracy and generalization of these models by using external datasets. As a result, the generalization of the automatic BAA model on the target dataset declines during test, which is mainly attributed to covariate shift (P(x) varies) [12] and prior probability shift (P(y) varies) [13]. For P(x) varies, the image distributions of the source and target data vary because of the use of different X-ray machines, image transmission protocols, and image shooting modes. The variations of P(y) are mainly caused by the different growth and development stages of hand bone characteristics of patients from different races, and the “Ground truth” of images is labeled by different standards. For instance, Westerners and Easterners adopt inconsistent GP atlas, and the TW method adopts different standards for the grade labeling of each ROI for different races. Therefore, the BAA model requires a re-collected target dataset and fine-tuning based on transfer learning to improve its generalization among different races. However, this suffers from the following challenges: (1) It is hard to acquire enough accurately labeled images since many professional radiologists and material resources are needed. (2) In the radiological report, bone age is generally expressed by uncertain description or an interval, such as “approximately 12 years old”, or “aged between 13 and 14”. In comparison with the accurate labeling to months in the RSNA dataset, only ambiguous bone age labels can be obtained. (3) The multi-stage training method needs to annotate the ROIs of hand bones, resulting in extra workload. To address the above challenges, we propose an automatic BAA method based on multi-branch attention learning with ambiguous labels (MAAL-Net). Specifically, this method extracts ambiguous labels of bone age from the radiological report by using natural language processing (NLP) techniques, acquires ROIs of hand bone through the multi-branch attention learning mechanism, and predicts bone age through end-to-end training.

The major contributions of this study are as follows: (1) A novel MAAL-Net is proposed, which automatically extracts hand object and bone part regions with only image-level bone age annotation. Our method is very similar to clinical assessment techniques, which makes it transparent for clinicians. (2) Considering the high difficulties in collecting enough accurately labeled bone age data, ambiguous labels are extracted from the radiological report by using NLP techniques. These ambiguous labels are used to train the model for automatic and accurate bone age prediction. (3) To the best of our knowledge, this study is the first to train, verify, and test the automatic BAA method on datasets of different races, and it will promote the applications of automatic BAA systems worldwide.

The rest of this paper is organized as follows. The related work is reviewed in Section 2. Section 3 illustrates the details of MAAL-Net. Section 4 presents the experiments and shows the experimental results of BAA task. The conclusion is given in Section 5.

## 2. Related Work

Automatic BAA methods consist of three main categories. The first category is the traditional machine learning method. Some traditional methods use hand-crafted features, e.g., gray-level co-occurrence matrix (GCLM) features, local binary pattern (LBP) features, and scale-invariant feature transform (SIFT) features, to train support vector machine (SVM) or its deformation methods [14,15,16]. Besides, BoneXpert [17] is known as the most popular automatic BAA software in Europe. It carries out automatic BAA by shape-driven active appearance model and predicts bone age features extracted by the principal component analysis method. The rejection rate of poor-quality images is approximately 1%, while the accuracy of this system is around 7.8 months. In general, traditional machine learning methods employ hand-crafted features that are sensitive to image quality and have poor accuracy.

The second category adopts the ROI-free method with single-stage structure to predict bone age, which uses the whole image as the input. For example, Larson et al. [18] used ResNet50 as the backbone network and 14,036 X-ray images of hands as the training set. The network determines the bone age according to the probability for every X-ray image belonging to which month. The mean absolute error (MAE) of this network on the test set was 6.24 months. Spampinato et al. [19] proposed and tested several DL-based methods for automatic BAA. Results demonstrated an average variation between automated and manual assessments of approximately 0.8 years. Recently, Cristina et al. [20] proposed a specific identity labels-based bone age assessment (SIMBA) model, which involved the gender as well as the age of patients. Different from the other bone age assessment methods, this model calculates the relative bone age according to the difference between practical age and bone age. The SIMBA proposes a new paradigm for BAA and achieves an MAE of 5.47 months on the private dataset RHPE. However, the application of this model is limited by the facts that a patient’s age is private and medical institutions usually do not provide age data to the public. All these methods predict bone age by training end-to-end deep neural networks using the entire image as input, which results in limited accuracy and poor interpretability.

The third category adopts a ROI-based method with the multi-stage structure to predict bone age, which extracts local information and ultimately predicts bone age by introducing prior knowledge for image segmentation or hand bone ROIs detection. For instance, Escobar et al. [21] proposed the Bonet which introduced a hand detection module and pose estimation module to the BBA system. This method had an MAE of 4.14 months on the RSNA dataset by virtue of its fine granularity and precisely localized annotation. Wang et al. [22] estimated bone age by extracting the local regions of phalanges and ulna from hand images based on RCNN. However, this model only evaluated the performance of local regions. Wang et al. [23] proposed the anatomical local-aware network (ALA-NET), which learned the architecture of hands and extracted local features following the spirit TW2 method. They estimated bone age by analyzing the features of ROIs, and this model obtained an MAE of 4.49 months without ROIs annotations. Chandran et al. [24] proposed to use the segmented ROI as input to a Generative Adversarial Network (GAN) for bone age prediction. However, GAN gradient descent sometimes fails to reach Nash equilibrium, resulting in unstable training, vanishing gradients, mode collapse, and other issues. Although GANs can achieve excellent performance, this method still requires a dataset with accurately annotated ROIs. Additionally, the model is trained on a bone age dataset of Western children, which is very different from Eastern children in terms of growth and development patterns and bone age assessment standards. Therefore, this trained model cannot be directly used for bone age assessment in Eastern children. Although the multi-stage structure achieves better performance, these models have high complexity and long computational time due to the need for prior knowledge and extra annotations.

In contrast to the above studies, the proposed MAAL-Net overcomes the difficulty in acquiring exact labels by using ambiguous labels. Moreover, it enables an end-to-end neural network to detect the hand object and the most informative bone part regions without using bounding box or part annotations.

## 3. Methodology

**Figure 1:**
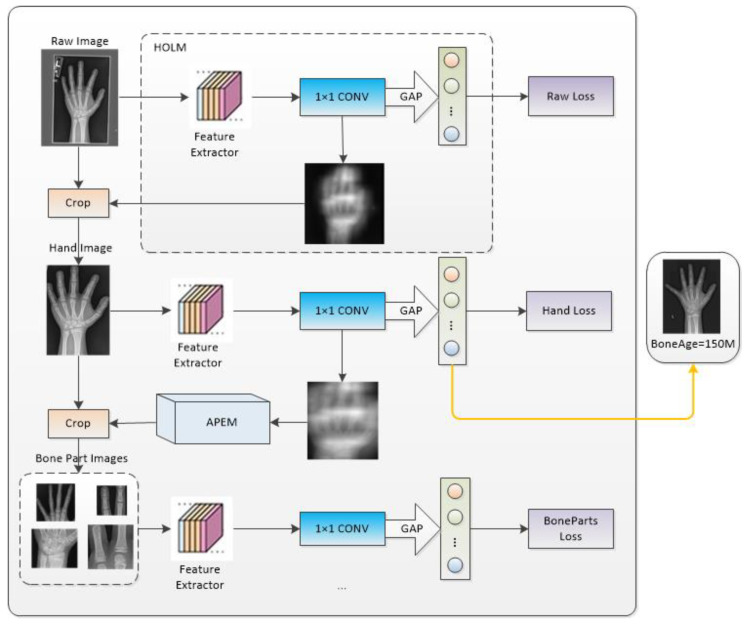
Structure of Multi-branch Attention Learning with Ambiguous Label Network (MAAL-Net).

As shown in Figure 1, the proposed MAAL-Net has three branches in the training stage: the raw image branch (Branch 1), the hand image branch (Branch 2), and the bone part images branch (Branch 3). Through the collaborative learning of these three branches, the network achieves a good classification ability for different scales and parts of objects as well. In the test phase with repeat experiments, Branch 2 achieves the best performance. Accordingly, we use Branch 2 as the final prediction output. Two modules are included among the three branches. One is the hand object location module (HOLM) to predict the position of the hand object, and the other is the attention part extraction module (APEM) to extract the ROIs with the most information of the hand object without using bounding-box annotations. Specifically, HOLM uses the whole image as input and generates the hand object that covers the most discriminative regions. This image is then used as the input of APEM to further crop bone part regions for training.

### 3.1. Construction of Ambiguous Label

Existing BAA methods adopt strong supervision hypotheses during learning and modeling: sufficient labeled samples are available during training, and the label of each sample is unique and explicit. Nevertheless, precise month-to-month bone age labels cannot be obtained in practical clinical diagnosis reports due to the inconsistent diagnostic criteria and professional levels of expertise. Common labels are ambiguous labels, such as bone age of approximately 12 years old or 12–13 years old. In other words, physicians can exclude impossible bone age, but they cannot provide an accurate and explicit bone age from several similar values. Hence, such ambiguous labels should be transformed into weak labels for learning. In the present study, ambiguous labels are transformed into label distribution.

**Figure 2:**
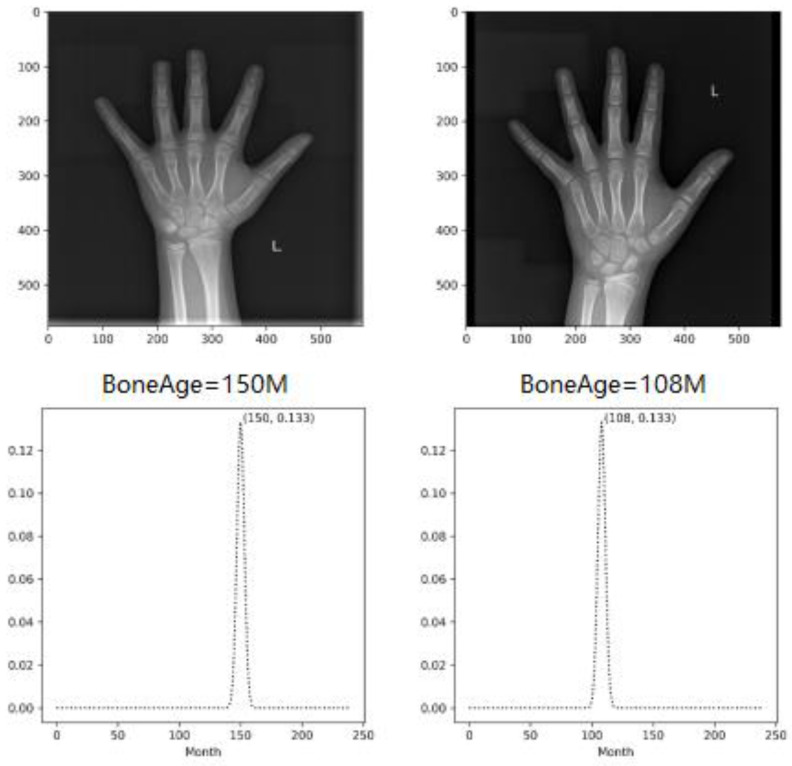
Bone age distributions.

Label distribution learning [25]: the real label distribution (y) is usually unavailable for an ambiguous label, which should be generated under an appropriate hypothesis. The label distribution $y=(y_{1},y_{2},...y_{C})$ must satisfy the following two conditions: (1) y shall be the probability distribution, $y_{i}\in [0,1]$ and $\sum_{i=1}^{C}y_{i}=1$ where c is the number of the classes. (2) The probability value ($y_{i}$) related to images varies among all possible labels. Relatively certain categories shall be vested with higher probability values, while those uncertain categories shall be given with relatively lower probability values. For BAA, the hypothesized probability shall be concentrated around the bone age labeled by physicians. Hence, ambiguous labels are quantified by Gaussian distribution. For bone age, a target label set can be found, where 0 < Bone-age ≤ 240. It is quantified into an integral ordered label set *L* = [*l*_1_, *l*_2_,…, *lc*], where *l_i_* is the possible prediction for y. y = { $y_{1}$, $y_{2}$, $y_{3}$…, $y_{C}$}, where $y_{i}$ is the probability that y = *l_i_*. Considering that equal step length is used in the quantization of y, the distribution y is generated by the following Gaussian probability density function:

(1) $$y_{i}=\frac{p(l_{i}|\mu ,\sigma )}{\sum_{C}p(l_{c}|\mu ,\sigma )}$$

where $p(l_{i}|\mu ,\sigma )=\frac{1}{\sqrt{2\pi }\sigma }\exp(-\frac{{\left(l_{i}-\mu \right)}^{2}}{2\sigma^{2}})$, p is the probability density function and *σ* is the standard deviation. We analyze the hyper-parameter *σ* in Section 4.4, and set *σ =* 1, 2, 3, 4 respectively in this study. The label distributions of images with bone age labeled at approximately 9 years old (~108 months) and labeled at 12–13 years old (~150 months) are shown in Figure 2.

### 3.2. Hand Object Location Module (HOLM)

Given an input image, we first use a feature extractor to obtain representative features $F\in R^{H\times W\times K}$, where K refers to the channel dimension, and H×W refers to the feature resolution. *F* is then used as the input of a 1×1 convolutional layer to obtain the feature map $A\in R^{H\times W\times C}$. Specifically, the *c*-th (c≤C) channel of A is expressed as

(2) $$A_{c}\left(i,j\right)=\sum_{k=0}^{K-1}W_{kc}F_{ijk}$$

where $F_{ijk},i\in R^{H},j\in R^{W},k\in R^{K}$ presents each pixel value of the channel k in F. $W_{kc}$ presents the weight of the *k*-th input neuron and the *c*-th output neurons in the 1×1 convolutional layer. Afterward, A is fed into a pooling layer [26]. In this study, we use the global average pooling to obtain the output $\hat{y}\in R^{C}$,

(3) $${\hat{y}}_{c}=\frac{1}{H\times W}\sum_{ij}\sum_{k=0}^{K-1}W_{kc}F_{ijk}$$

where $c\in R^{C}$ is the label category, and ${\hat{y}}_{c}$ denotes the probability of category *c*. Obviously, ${\hat{y}}_{c}$ is the global pooling result of $A_{c}$. That is,

(4) $${\hat{y}}_{c}=\frac{1}{H\times W}\sum_{ij}A_{c}\left(i,j\right)$$

In a word, the activated output A of the final convolutional layer (1×1 convolutional layer) for the input image labeled *c* could be obtained through HOLM. Specifically, $A_{c}$ of the *c*-th channel can reflect the impact of each pixel on the ultimate prediction results. When global average pooling is applied, the classification results are only sensitive to the peak value of $A_{c}$. Under such circumstances, the region where peak value of $A_{c}$ is located is regarded as the most discriminative region, which is called ROI.

Considering that labels are ambiguous labels in Gaussian distribution, and the probability sum of all categories is 1, the output ($\hat{y}$) needs to be transformed into the Gaussian distribution *p* by using the Softmax activation function. The *p*-value is formulated as

(5) $$p_{c}=\frac{\exp({\hat{y}}_{c})}{\sum_{c=0}^{C-1}\exp({\hat{y}}_{c})},c=0,1,...,C-1$$

where $p_{c}$ is positively related to ${\hat{y}}_{c}$. Consequently, the effect of the peak values of $A_{c}$ on ${\hat{y}}_{c}$ are positively related to their effect on $p_{c}$.

(6) $$P_{c}\propto \frac{1}{H\times W}\sum_{ij}A_{c}\left(i,j\right)$$

Finally, after the normalization of $A_{c}$, we design a binary mask M to pinpoint the most discriminant areas:

(7) $$M_{c}\left(i,j\right)=\left\{\begin{array}{c} 1\;\;\;\;A_{c}(i,j)\geq \tau \\ 0\;\;\;\;A_{c}\left(i,j\right)<\tau \end{array}\right.$$

where τ is the threshold size of RoI. When τ decreases, RoI becomes relatively large. Otherwise, the RoI becomes relatively smaller. Hence, a suitable RoI can be obtained by adjusting τ. In this study, we empirically set τ = 0.6.

### 3.3. Attention Part Extraction Module (APEM)

Since BAA is fine-grained, the accuracy is theoretically improved if bone part regions could be detected for training. In APEM, the region proposal network (RPN) [27] is used to generate the RoIs bounds ($R_{i}$) and the confidence score ($S_{R_{i}}$). $R_{i}$ was arranged in descending order according to $S_{R_{i}}$ and the top N regions $\left\{R_{1},R_{2},\ldots ,R_{N}\right\}$ are acquired using the non-maximum suppression (NMS) method [28].

Specifically, the output of HOLM serves as the input of APEM. For the activation output *A* of HOLM, we first unify its size to (576, 576) and then feed it into the RPN to produce a series of rectangle RoIs $\left\{R_{1},R_{2},R_{3},..,R_{M}\right\}$. The anchors of PRN are designed with ratios of {2:3, 1:1, 3:2} and scales of {128, 160, 224, 288, 384}. In other words, each point in *A* generates 3×5 groups of $R_{i}$. The normalization function is defined as

(8) $$z\left(x\right)=\frac{x-Min(x)}{Max\left(x\right)-Min(x)}$$

For the category *c*, we can obtain S by doing an exponential transformation on the feature map $A_{c}$, denoted as

(9) $$S\left(i,j\right)=z\left(\exp\left(\eta *z\left(A_{c}\left(i,j\right)\right)\right)\right),\eta >0$$

The hyper-parameter η controls the stretching degree of $A_{c}$. A higher η value indicates greater differences between each local peak and surrounding region. Under such circumstances, the mean of *S* under the coverage of each $R_{i}$ is used as the corresponding confidence score ($S_{R_{i}}$).

(10) $$S_{R_{*}}=\frac{1}{n}\sum_{i,j\in *}S(i,j)$$

In comparison with $A_{c}$,S after stretching can effectively assure that the confidence core of $R_{i}$ at peak is higher than others, and $R_{i}$ will not be eliminated during NMS, thus improving the reliability of location performances of APEM. Finally, $R_{i}$ is arranged in descending order according to confidence score:

(11) $$S_{R1}\geq S_{R2}\geq S_{R3}\geq \ldots \geq S_{RM}$$

The top *N* terms are used as outputs, which are $\left\{R_{1},R_{2},R_{3},..,R_{N}\right\}$ and ${\{S}_{R1}{,S}_{R2}{,S}_{R3},\ldots {,S}_{\mathrm{RN}}\}$. Finally, the crop regions are unified to (288, 288) by bilinear interpolation.

### 3.4. Loss Function

Cross-entropy loss is widely used in convolutional neural network-based classification tasks. However, this work transforms ambiguous labels into label distribution for learning, resulting in the inability to apply cross-entropy loss for BAA learning directly. To train the network while making the ambiguous labels work effectively, we propose the loss functions $L_{\mathrm{raw}}$ and $L_{\mathrm{hand}}$ for the raw and hand image branch, respectively.

(12) $$L_{\mathrm{raw}}=L_{\mathrm{KL}}(Y,{\hat{Y}}_{raw})+L_{mae}(Y,{\hat{Y}}_{raw})+T(t).L_{t}({\bar{Y}}_{raw},{\hat{Y}}_{raw})$$

(13) $$L_{\mathrm{hand}}=L_{\mathrm{KL}}(Y,{\hat{Y}}_{hand})+L_{mae}(Y,{\hat{Y}}_{hand})+T(t).L_{t}({\bar{Y}}_{hand},{\hat{Y}}_{hand})$$

where ${\hat{Y}}_{\mathrm{raw}}$ and ${\hat{Y}}_{\mathrm{hand}}$ are the output results of the last Softmax layer, whose distributions are consistent with the definition of *Y*. In other words, ${\hat{y}}_{i}\in [0,1]$ and $\sum_{i=1}^{m}{\hat{y}}_{i}=1$. ${\bar{Y}}_{\mathrm{raw}}$ and ${\bar{Y}}_{\mathrm{hand}}$ are the values produced by assembled prediction, which are updated in each epoch. *T*(*t*) is a time-dependent weighting function. For the raw and hand image branch, Equation (12) is used as the loss function. The bone part image branch does not use temporal ensembling as the third term of loss function because of the great difference in the prediction values of each RoI. In other words, $L_{\mathrm{bonePart}}=L_{\mathrm{KL}}(Y,{\hat{Y}}_{\mathrm{bonePart}})+L_{mae}(Y,{\hat{Y}}_{bobePart})$. Specifically, the first term of loss function aims to calculate the relevance between the predicted bone age distribution ($\hat{Y}$) and the ground-truth bone age distribution (*Y*). Different methods can be used to estimate the relevance between two distributions, e.g., Kullback–Leibler (KL) divergence is adopted in this study, as Equation (14) defines:

(14) $$L_{\mathrm{KL}}(Y,\hat{Y})=-\frac{1}{m}\sum_{i=1}^{m}y_{i}^{T}\log(\frac{y_{i}}{{\hat{y}}_{i}})$$

The second term of the loss function aims to calculate the MAE loss between $\hat{Y}$ and *Y*, defined as Equation (15):

(15) $$L_{\mathrm{mae}}(Y,\hat{Y})=\frac{1}{m}\sum_{i=1}^{m}|y_{i}-{\hat{y}}_{i}|$$

The third term of the loss function is the temporal ensembling term. It makes $\hat{Y}$ at present training epoch consistent with the network output results ($\bar{Y}$) in different epochs. Based on the temporal ensembling [29] used for semi-supervised learning, we assemble model predictions in different epochs as auxiliary supervision information for the next epoch. In comparison with the current predictions, the training targets generated by the assembled prediction might be closer to the ground-truth label distribution. Therefore, they can be used to guide network training. The temporal ensembling loss function is defined as Equation (16):

(16) $$L_{t}(\bar{Y},\hat{Y})=-\frac{1}{m}\sum_{i=1}^{m}{\bar{y}}_{i}^{T}\log(\frac{{\bar{y}}_{i}}{{\hat{y}}_{i}})$$

where ${\bar{Y}}_{i}$ is the assembled predictions generated in each epoch. We use the exponential moving average (EMA) of predictions in every epoch to generate the assembled predictions. The assembled predictions S and the training target $\bar{Y}$ are updated as follows:

(17) $$S^{t}=\gamma S^{t-1}+(1-\gamma ){\hat{Y}}^{t}$$

(18) $${\bar{Y}}^{t+1}=S^{t}/(1-\gamma^{t})$$

where $S^{t}$ and $S^{t-1}$ denote the assembled predictions at *t*-th and (*t* − 1)-th epochs, respectively. Coefficient γ is the momentum term. As expressed in Equations (17) and (18), EMA assigns smaller weights to early prediction and increases the weights as the training epoch increases. Considering that prediction values are not present before the first epoch, $\bar{Y}$ and S are initialized as 0.

The loss function has the term *T*(*t*), which is a time-dependent weighting function and increases with the number of epochs. The prediction results are unreliable at the beginning phase of training because the network has not been trained well. As a result, the assembled prediction can only provide very limited supervision information. As the training goes on, the prediction ability of the network is strengthened gradually, and the assembled prediction can guide the network to obtain better and more accurate results. *T*(*t*) is defined as follows:

(19) $$T(t)=T_{\max}\cdot \exp(-5{\left(1-t\right)}^{2})$$

where *T_max_* is the maximum value that *T*(*t*) can reach. With the increase of time *t*, *T*(*t*) increases from 0 to *T_max_* gradually.

Ultimately, the total loss function is expressed as:

(20) $$L_{total}=L_{raw}+L_{hand}+L_{boneParts}$$

## 4. Experiments and Results

### 4.1. Datasets and Evaluation Metric

The RSNA dataset [30] was proposed by Radiological of North America in the machine learning challenges of pediatric bone age. The RSNA dataset is composed of 12,043 X-ray images of children’s left hands which are interpreted by pediatric radiologists and recorded in the radiology report for bone age [3]. Among them, 9634 images are utilized for training, 1204 images are used for validation, and the final 1205 images are used for testing. The average patient age in the training and validation set is 127 months, while that in the test set is 132 months.

X-ray images in the CNBA dataset are obtained from Chinese children. Unlike the other BAA datasets mainly composed of Westerners, East Asians dominate the CNBA dataset. The CNBA dataset has 46% male and 54% female X-ray images, with patient ages ranging from 0 to 240 months. Ambiguous labels are extracted by NLP techniques from clinical radiological reports, including bone age and gender. The CNBA dataset contains 4526 images, of which 3620 images are utilized for training, 452 images for validation, and 453 images for testing.

The MAE between predicted bone age ${\hat{y}}_{i}$ and its ground truth $y_{i}$ is used as the performance evaluation metric, defined as

(21) $$\mathrm{MAE}=\frac{1}{N}\sum_{i=1}^{N}\left|y_{i}-{\hat{y}}_{i}\right|$$

Another evaluation method follows [31] to calculate the accuracy within 6 months, 12 months, and 24 months.

### 4.2. Implementation Details

The implementation of MAAL-Net is very flexible. In the present study, we implement the backbone of MAAL-Net using ResNet50, which can also be replaced by other networks, such as VGG, ResNet, and Inception. In the experiment, the input size is uniformly 576 × 576, and the batch size is 32. Comparative experiments are conducted with *σ* ranging from 1 to 4. Finally, Branch 2 achieves the best performance when *σ* = 3. MAAL-Net is implemented by Pytorch and is trained on a CentOS workstation with four NVIDIA GTX 1080Ti 12G GPU. Momentum SGD is chosen as the optimizer with an initial learning rate of 0.001 and declined by a factor of 10 per 30 epochs. Finally, the model that performed best on the validation set is chosen for testing.

### 4.3. Experimental Results

In this section, we compare our MAAL-Net with the state-of-the-art ones. The results are listed in Table 1. Generally, the RoI-based methods [21,32,33] that detect the local regions as RoIs by annotating additional bounding boxes or key points achieve better performance. By contrast, the RoI-free methods [18,19,34,35] that use entire images as input are inferior because they only use global features. However, these RoI-free methods can save a lot of manpower and materials for annotations. The method in [36] predicted bone age by using a two-stage network, which does not require ROI annotation yet achieves comparable performance as the ROI-based method. However, this method suffers some limitations because it cannot apply end-to-end training. The proposed MAAL-Net uses the multi-branch attention learning network without bounding boxes or part annotations. In comparison with RoI-free methods, the presence of multi-branch attention gives MAAL-Net a significant improvement in prediction accuracy. In comparison with RoI-based methods, the MAAL-net automatically determines the most informative regions without bounding boxes or part annotations. In comparison with the previous method [36], the MAAL-Net allows end-to-end training in a short inference time without extra processing. The experimental results indicate that our method yields state-of-the-art performance with MAE = 4.09 on the RSNA dataset.

To our knowledge, MAAL-Net is the first to train a network with ambiguous labels, which extracts ambiguous labels directly from radiological reports as training and test sets. Considering that the bone images in the CNBA test set are labeled by an ambiguous value or interval, the accuracy of MAAL-Net is evaluated based on the deviation between the predicted and the ground-truth bone age within ±6 months, ±12 months, and ±24 months. The results are shown in Table 2. Thanks to the label distribution learning that prevents over-fitting, the accuracy of the method is significantly improved by 61% within ±6 months, 7.8% within ±12 months, and 1.43% within ±24 months compared to the method in [37]. Moreover, we further test our MAAL-Net on CNBA dataset, achieving an accuracy of 87.27% within ±6 months and 96.65% within ±12 months, which are only approximately 2% lower than those on the RSNA dataset. The accuracy within ±24 months of the proposed method reaches 99.36%, which is close to 100%. In comparison with the MAE of human experts in clinical bone age diagnosis with a value of 7.32 months [18], the accuracies within ±6 months of MAAL-Net on two datasets are 89.14% and 87.27%, which meet the expectations for clinical bone age diagnosis.

### 4.4. Analysis of Hyper-Parameters

Ablation experiments are performed on ambiguous labels and multi-branch attention structures. To analyze the effect of hyper-parameter *σ*, MAAL-Net is tested several times for *σ* values ranging from 1 to 4. As shown in Table 3, MAAL-Net obtains the highest accuracy with MAE = 4.07 when *σ* = 3. We can find that a suitable *σ* can effectively reduce the MAE. For the Gaussian distribution, the area in the horizontal axis interval (−2.58*σ*, +2.58*σ*) is 99.730020%. When *σ* = 3, we obtain 2.58*σ* = 7.74, which is close to the MAE = 7.32 of human experts in clinical bone age diagnosis using the Greulich and Pyle approach [18]. Therefore, it is a good choice to set *σ* = 3. As we can see, the lowest MAE performance is simply obtained from the hand image branch which is used as the final prediction output during the test phase.

### 4.5. Visualization and Interpretation

**Figure 3:**
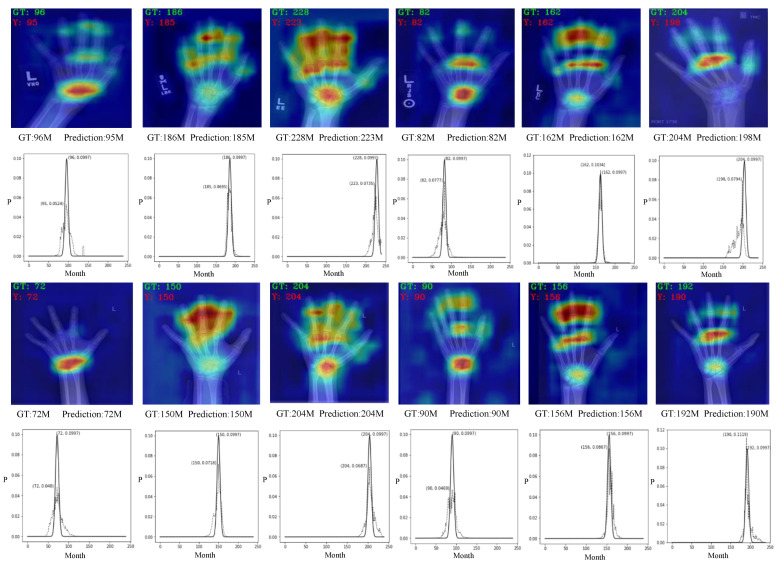
Visualization of bone age prediction results. Rows 1 and 3 show the attention heat maps of the RSNA dataset and CNBA dataset, respectively. Rows 2 and 4 show the bone age prediction results of X-ray images from the RSNA dataset and CNBA dataset, respectively. The solid line represents the ground-truth label transformation into Gaussian distribution, while the dotted line represents the label distribution output by the model prediction.

We further perform a visualization experiment to effectively and intuitively understand the proposed method. Heat maps generated by Grad-CAM [38] on input images are shown in rows 1 and 3 of Figure 3. The peaks of heat maps indicate that the important features are mainly concentrated in the most distinctive parts, such as the carpal and phalanges. For young children, the important features are concentrated on the carpal rather than the phalanges, as shown in columns 1 and 4 in Figure 3. For teenagers and those in late adolescence, important features are concentrated at the carpal and phalanges, and the features of the carpal are stronger than those of the phalanges, as shown in columns 2, 3, 5, and 6 in Figure 3. These findings are consistent with prior knowledge of experts for BAA. Therefore, the proposed method allows us to learn and establish accurate knowledge without prior knowledge of experts. Furthermore, we provide the distribution curves of the predicted output and ground-truth labels as shown in rows 2 and 4 in Figure 3. As we can see, most of the prediction and ground-truth label distribution curves have very strong consistency. important features are concentrated in the wrist and toe bones, but the features of the wrist bones diminish while those of the toe bones increase, as shown in columns 2, 3, 5, and 6 in Figure 3.

## 5. Conclusions

This study presents a novel automatic BAA method MAAL-Net based on multi-branch attention with ambiguous labels. This method addresses the lack of accurately labeled images with ROI annotations from different races by extracting ambiguous labels using NLP techniques from radiological reports for training. In particular, our method solves the problem that existing models cannot be used for the BAA of Eastern children. The proposed MAAL-Net makes effective use of the ambiguous labels in feature and classifier learning, and the multi-branch attention structure can fully learn global and local features to achieve excellent performance. Compared to methods that require accurately labeled datasets, our approach achieves competitive results. Notably, the accuracy of our method within ±6 months is significantly improved compared to previous studies. These results demonstrate the potential of our model to be applied to the BAA of Chinese children, reducing the workload of experts and providing a reference for routine screening of children’s growth and development. This study also provides a feasible solution for effective medical imaging learning by exploring medical knowledge from radiology reports in the future.

## 6. Limitations and Future Directions

Although much effort has been made on the automatic BAA system, it still suffers from some limitations to be further investigated.

First, the dataset is annotated at a relatively coarse granularity. The X-ray image in the dataset has only one coarse label and there is no fine-grained label for the ROI to determine bone age. This makes the network produce a deviation when extracting the key features of the ROI, which further leads to a more serious error in the final classification of the bone age. Due to the limitations of data annotation, the model training in this paper is based on the characteristics of the whole hand bone using the GP atlas method. Although the GP atlas is simple, intuitive, and fast, its prediction accuracy of bone age is inferior to the later TW3 method and CHN05 method. In the follow-up study, we will continuously improve the dataset by labeling 13 different ROI of hand bones and giving the development level of each bone part. For the bone age prediction methods, TW3 and CHN05 methods with higher accuracy will be adopted. For the backbone network, we will use the transformer-based DETR [39] (Detection Transformer) to improve the accuracy of hand bone ROI positioning and enhance interpretability. The accuracy of bone age prediction will also be further improved.

Secondly, there is an imbalance in data categories. The experiment in this paper is conducted on hand images of children aged 37–228 months. The large span of bone age and the significant differences in the data volume of each age group, especially the relatively small number of bone age images in late adolescence, lead to the different accuracy of bone age prediction for different age groups in the test phase. In the follow-up study, we consider conducting independent training for different age groups, such as preschool, school-age, and adolescents. Furthermore, new loss functions are supposed to train the network. For example, we will use LMFLOSS [40] function to dynamically consider hard samples and class distribution and alleviate the poor performance caused by data class imbalance.

Finally, there is the limitation of domain shift. The X-ray image of patients’ hands are obtained from different machines and equipment in multiple hospitals. The scanning protocol, filming parameters, shooting angle, and subject group are different, which leads to poor generalization performance in the subsequent tests in different hospitals. In future research, we will focus on the deep feature representation of data to capture more information conducive to the bone age prediction task, select the closest two domain samples, close the same category distance between the two domains, and widen the distance between different categories, to achieve cross-domain similarity and dissimilarity, improve bone age prediction precision, and make it have better generalization ability and stability.

## Figures and Tables

**Table 1:** Mean Absolute Error (MAE) results of different methods.

Methods	Dataset	RoI Annotation	Ambiguous Label	MAE (M)
Human Expert et al. [18]	RSNA	-	-	7.32
Larson et al. [18]	RSNA	NO	NO	6.24
Spampinato et al. [19]	Private	NO	NO	9.12
Iglovikoc et al. [32]	RSNA	YES	NO	4.97
Tom et al. [34]	RSNA	NO	NO	6.8
BoNet [21]	RSNA	YES	NO	4.14
AR-CNN [33]	RSNA	YES	NO	4.38
Chen et al. [36]	RSNA	NO	NO	4.7
Pan et al. [35]	RSNA	NO	NO	7.35
SIMBA [27]	Private	NO	NO	5.47
MAAL-Net [ours]	RSNA	NO	YES	4.09

**Table 2:** Accuracy results of different methods.

Methods	Dataset	±6 Months	±12 Months	±24 Months
Hao et al. [37]	RSNA	55.28%	91.09%	98.50%
MAAL-Net [ours]	RSNA	89.14%	98.24%	99.93%
	CNBA	87.27%	96.65%	99.36%

**Table 3:** Ablation study on the construction of ambiguous labels and multi-branch attention on RSNA dataset.

Hyper-Parameter	Raw Image Branch	Hand Image Branch	Bone Part Images Branch
*σ* = 1	5.92	5.1	7.54
*σ* = 2	5.72	4.87	6.53
*σ* = 3	4.86	4.07	5.68
*σ* = 4	5.29	4.9	5.96

## Data Availability

https://www.kaggle.com/kmader/rsna-bone-age (accessed on 21 March 2023).
